# The Dark Web of Machiavellianism and Psychopathy: Moral Disengagement in IT Organizations

**DOI:** 10.5964/ejop.4011

**Published:** 2022-05-31

**Authors:** Alexandra Maftei, Andrei-Corneliu Holman, Alina-Georgiana Elenescu

**Affiliations:** 1 Faculty of Psychology and Education Sciences, Alexandru Ioan Cuza University of Iaşi, Iaşi, Romania; Aristotle University of Thessaloniki, Thessaloniki, Greece

**Keywords:** organizational moral disengagement, IT, age, gender, ethical behavior

## Abstract

In the current paper, we were interested in examining a series of predictors of organizational moral disengagement, namely Machiavellianism and psychopathy, along with a series of demographic variables (i.e., gender, age, and work experience). Our sample consisted of 114 IT employees aged 21 to 54 (M = 28.51, 62% males). We used a cross-sectional approach and an original scale to measure organizational moral disengagement. The hierarchical regression analysis suggested that the most important predictor of organizational moral disengagement was Machiavellianism, followed by gender (i.e., males). A significant, negative association emerged between organizational moral disengagement and age, suggesting that the older we grow, the lower the organizational moral disengagement. Machiavellianism and psychopathy were significantly associated with all moral disengagement mechanisms, except one - diffusion of responsibility. The most powerful association we found were between Machiavellianism and moral justification and between psychopathy and euphemistic language. Theoretical and practical implications are discussed.

Organizational moral disengagement is, generally, a costly behavior to many companies and employers. While some predictors of immoral organizational behavior have already been identified, our aim was to add to our predictive capabilities by exploring a series of predictors and associations related to moral disengagement and its mechanisms within several IT Romanian organizations.

A recent study (Ernst & Young, 2017) suggested that 15% of Romanian employees would resort to immoral behavior to advance or to obtain a salary increase. Also, 30% of the interviewees mentioned that they were afraid to report the company's or a colleague's fraud because it would slow down their career advancement. However, almost a quarter of the respondents resigned after acknowledging immoral behavior at their workplace. Within the IT environment, immoral behavior may include online fraud, hacking of various databases (especially those containing with sensitive information, such as money spending), or collecting and using data to manipulate Internet users (e.g., the famous Facebook and Cambridge Analytica case, which proved that the latter company collected information from users of the Facebook platform without their consent, by displaying various advertisements intended to influence their vote in the presidential elections in the United). Additionally, one of the most unethical behaviors related to the IT domain is the creation and usage of *the dark web* (and, more recently, *the deep web*) – an online network that facilitates various crimes, from drug and arms sales to paid assassins and child pornography.

## Learning Our Moral (Dis)Engagement

Over time, several theories explained the human learning process, from classical conditioning (Pavlov, 1926), operant conditioning (Skinner, 1966; Thorndike, 1898), cognitive learning (Tolman, 1932), to social learning (Bandura, 1971). Bandura's paradigm emphasizes the role of the individual as the agent who influences the actions of those around him or the course of events through one takes action. The human agent manifests itself through prediction, reaction, and reflection. In the prediction phase, people are motivated and guided by action plans, goals and challenges, and, more importantly, by visualizing their efforts' potential results. This anticipatory behavior is governed by the visualization of goals and expected results rather than by the future's unfulfilled state. However, regulating behaviors that violate specific rules operates on a legal, social, and evaluative level, based on anticipated consequences. Therefore, people may refrain from violating the laws due to the fear of being caught and suffering the consequences of their transgressions (i.e., imprisonment) or the fear of social censorship and other social negative consequences.

The Moral Disengagement (MD) theory (Bandura et al., 1996) explains why individuals can engage in immoral behaviors without any apparent guilt or self-censorship. Most people have developed moral standards, which they use to anticipate and judge their behaviors. Actions that contravene these standards lead to guilt and self-condemnation. Typically, individuals behave ethically, following internal moral values, because they anticipate positive or negative self-assessments of potential behaviors. On the other hand, moral self-regulation can be activated and deactivated selectively so that, when inactive, using moral disengagement, people may be freed from the guilt and self-censorship that usually arise when they behave immorally (Qin et al, 2020).

Specifically, Bandura et al. (1996) described eight moral disengagement mechanisms, which serve to distance the individual from harmful behavior cognitively and avoid negative feelings, such as guilt or shame. These mechanisms can be grouped by location, depending on whether they target the behavior, agent, effects, or victim. In short, the mechanisms of moral disengagement referring to the locus of behavior explain how the individual considers negative and harmful behaviors to be more positive, as a result of *moral justification* (by restructuring negative actions into something socially acceptable), *advantageous comparison* (contrasting negative behavior with something more negative), or *euphemistic labeling* (using language to consider the immoral behavior as less harmful). The agent's locus includes mechanisms by which the individual minimizes his/her role and detaches from moral responsibility by either shifting responsibility (considering his/her behavior as a result of pressure from authority) or minimizing if others are involved). The mechanisms involving the locus of effects refer to disregarding or distorting the consequences of inhuman behavior. Finally, mechanisms implying the victim's locus refer to the cognitive processes by which feelings of guilt or remorse can be avoided either by dehumanizing or blaming the victim while also holding them accountable for his/ her suffering (Bandura, 2002; Bjärehed, 2020).

The organizational environment seems to provide multiple opportunities for moral disengagement: organizations tend to have a hierarchical scale, often providing the possibility to shift responsibility; work is often undertaken in a team, making it possible to spread responsibility; the organization generally defines the boundaries of its employees when it comes to their work behavior (Barbaranelli, 2004; Barsky, 2011), thus providing numerous opportunities for moral justifications (e.g., "I did that to protect the organization") and minimizing the consequences of immoral behavior (Moore, 2012). The predilection for moral disengagement can be detrimental to both the individual and the organizational environment (Moore, 2008). Disengaged moral reasoning can occur and persist in almost all organizations, including those who make both formal and informal efforts to promote and support ethical behavior, and may lead to corruption, job insecurity, negative emotions, and general deviant work-behavior (Barsky, 2011; Fida, Paciello, Tramontano, Fontaine, Barbaranelli, & Farnese, 2015; Huang, Wellman, Ashford, Lee, & Wang, 2017; Martin, Kish-Gephart, & Detert, 2014; Moore, 2008; Samnani, Salamon, & Singh, 2014).

## Machiavellianism, Psychopathy, and Organizational Behavior

Undoubtedly, there is a particular fascination in exploring the characteristics of people who have antagonistic traits (Jonason, Webster, Schmitt, Li, & Crysel, 2012). The social adversity of the dark triad of personality caught both researchers and the general public's attention. Since its conceptualization (Paulhus & Williams, 2002), it has been extensively investigated in a high number of various scientific approaches (e.g., Glenn & Sellbom, 2015; Kaufman, Yaden, Hyde, & Tsukayama, 2019; Lyons, Houghton, Brewer, & O’Brien, 2022; Moraga, Nima, & Garcia, 2017; Sabouri et al., 2016; Szabó & Bereczkei, 2017; Wissing & Reinhard, 2019). The dark triad consists of three distinct personality traits, which can sometimes overlap: Machiavellianism, subclinical narcissism, and subclinical psychopathy. They all share common features, such as manipulativeness, insensitivity, and selfishness (Jones, 2013; Pilch & Turska, 2015) and the general indifference to social norms, which often leads to immoral behaviors (e.g., lying, deception, manipulation). Individuals who score high on the dark triad traits are considered untrustworthy and unromantic as relationship partners (Jonason, Li, & Czarna, 2013), cold parents, and obsessed with control, and they are often betraying their co-workers. In short, the dark triad has toxic consequences for those around people with these characteristics. On the other hand, the dark triad also has positive parts: especially in competitive situations (where they might win something), individuals with the features of the dark triad can be loyal friends, effective leaders, and heroic saviors (Lyons, 2019).

More importantly, the dark triad has been the subject of numerous studies within the organizational environment (e.g., Nübold et al., 2017; Palmer et al., 2017; Prusik & Szulawski, 2019; Wu, Wang, Zheng, & Wu, 2019). For example, Palmer et al. (2017) studied its impact on organizational behavior and the moderating role of perceived organizational support. Their results suggested that employees with elevated levels of narcissism or psychopathy tend to exhibit various counterproductive organizational behaviors (e.g., sabotage, abuse, theft, reduced production). Although the correlations between Machiavellianism and counterproductive behaviors were positive, a causal relationship between them could not be established. In terms of organizational support, the authors reported a positive, significant correlation with narcissism and a negative one with psychopathy. In a meta-analysis related to the link between the dark triad and counterproductive organizational behavior, Cohen (2016) suggests that there may not be a direct link between them, while others suggested their connection may actually be lying on moderation or mediation processes (Schyns, 2015).

We focused on two of the triad's personality traits within the current study, namely, Machiavellianism and psychopathy. Though previous studies explored narcissism (i.e., the third dark triad dimension) and its link to unethical behavior, results generally suggested more significant associations between moral disengagement and Machiavellianism and psychopathy, respectively, as important psychological antecedents to unethical behavior (e.g., Egan, Hughes, & Palmer, 2015). Additionally, narcissism was suggested to be differently related to moral disengagement compared to Machiavellianism and psychopathy (Sijtsema, Garofalo, Jansen, & Klimstra, 2019), as several studies identified different mechanisms of action concerning immoral behavior related to the latter two (e.g., Roeser, McGregor, Stegmaier, Mathew, Kübler, & Meule, 2016), at various age stages.

*Machiavellianism* is a personality trait characterized by a manipulative interpersonal style and the willingness to exploit others (Brewer & Abell, 2017). Machiavellian individuals are usually cynical, manipulative, secretive, and suspicious, avoiding norms and lacking empathy, who usually follow utilitarian models (Christie & Geis, 1970; Ináncsi, Láng, & Bereczkei, 2015; 2016; Jaffé, Greifeneder, & Reinhard, 2019). Researchers also hypothesized that Machiavellianism might not be qualitatively different from psychopathy but a less severe sub-clinical manifestation of it (e.g., Mealey, 1995). Within the organizational environment, Machiavellians usually hold leading positions, from which they manage to manipulate and control others. They are less willing to adhere to rules and procedures, focusing on their power over those around them (D’Souza, 2015). Machiavellians are usually sensitive to the social and organizational context and may change their tactics, from cooperation to competition, when necessary (Czibor, 2012). Sometimes, they engage and support emotional manipulation, to the point they manage to put other people against each other (Austin, 2007). They are more likely to lie during hiring interviews to get a job, compared to psychopaths or narcissists, and rarely show humility (Levashina & Campion, 2006; Roulin & Bourdage, 2017). Machiavellians may also spread rumors about their co-workers, hide important work information, or find subtle ways to denigrate his/her organizational team member (Greenberg & Baron, 2013). However, despite its harmfulness, Machiavellians are also distinguished by flexibility in the use of various strategies, from withdrawal to cooperation (depending on the context), to gain various personal benefits.

*Psychopathy* is, perhaps, the darkest component of the triad, and the reason behind lies within the strong association with indifference to others and the manifestation of disruptive interpersonal behaviors, such as bullying and sadism (Mathieu, Neumann, Babiak, & Hare, 2015 Paulhus & Williams, 2002). Ignorance of others is all the more evident in the context of breaking the law. Individuals with elevated levels of psychopathy are more likely to violate social and legal norms and to engage in immoral activities ranging from minor daily abuse to a lifestyle based on illegal activities, leading to incarceration and high levels of recurrence (Lyons, 2019). Psychopathy is marked by egocentrism, impulsivity, and lack of moral emotions such as guilt and regret. Psychopaths usually hold the ability to influence and dominate those around them; they generally have low levels of anxiety and engage in risky behaviors (LeBreton, Binning, & Adorno, 2006). Individuals high in psychopathy are thoughtless, selfish, aggressive, profiteering, and insensitive, usually blaming others or victimizing themselves. They are present-oriented and usually act without planning (D’Souza, 2015). Psychopathy encompasses other dimensions, such as impulsivity, risk-taking, and limited empathy for others (Ali, Amorim, & Chamorro-Premuzic, 2009). Individuals with high levels of psychopathy are usually more opportunistic and less flexible than those with Machiavellianism.

Within the organizational environment, many managers (and those in leading positions, in general) present the general features of psychopathy (ten Brinke, Kish, & Keltner, 2018; Spurk, Keller, & Hirschi, 2016). Psychopathic managers lead their organizations through power, money, and prestige and are indifferent to their colleagues' or employees' fate, generating a general toxic working environment (Hogan, Hogan, & Kaiser, 2011). They are opportunistic, self-centered, cruel, and cynical, but at the same time, they can be charming, manipulative, and ambitious. However, they usually jeopardize the business's performance and longevity because they put their interests above those of the organization. Moreover, the sense of social responsibility is threatened by the lack of guilt, shame, or regret about their immoral deeds (Boddy, 2005). As managers, individuals high in psychopathy were also found to be lacking relationship skills, being unable to build effective working teams (Hogan, Hogan, & Kaiser, 2011), and usually determining subordinates' stress, work-family conflicts, and low levels of job satisfaction, and counterproductive work behavior (Mathieu et al., 2015; O’Boyle et al., 2012).

## Aims of the Present Research

The present research aimed to explore a series of predictors of organizational moral disengagement. More specifically, we were interested in finding whether age, gender, education level, work experience, Machiavellianism, and psychopathy predict organizational moral disengagement within the IT work sector. Previous research suggested that men are generally more predisposed to disengage morally, compared to women (e.g., Clemente, Espinosa, & Padilla, 2019; Samnani et al., 2014; Wang et al., 2016), and that females generally have a more ethical work behavior (e.g., Chonko & Hunt, 1985; Fritzsche, 1988). Research on adolescents suggested that moral disengagement may decrease with age due to maturation and the internalization of moral values and social adjustment (Paciello, Fida, Tramontano, Lupinetti, & Caprara, 2008). However, other studies claimed the opposite (Wang, Ryoo, Swearer, Turner, & Goldberg, 2017).

Therefore, we assumed that the series of proposed predictors would significantly predict moral disengagement within the IT sector. More specifically, our hypotheses were the following: (a) age, gender, education level, work experience, Machiavellianism, and psychopathy would predict organizational moral disengagement within the IT work sector; (b) preliminary correlational analyses would suggest that men present more elevated organizational moral disengagement levels than women; (c) younger participants would be more likely to express morally disengaged organizational behavior, compared to older ones; and, finally, (d) in line with previous findings (e.g., Roozen, De Pelsmacker, & Bostyn, 2001), we also considered that participants with limited work experience would score less on the organizational moral disengagement scale. An important clarification is needed concerning the last two assumptions: within the IT sector, generally dominated, in Romania, by relatively young employees, compared to the mean European age (Eurostat, 2017), older participants do not necessarily have extended work experience.

The novelty of the present study lies in both the proposed association of predictors, as well as the original version of the scale that we used to measure organizational moral disengagement. Thus, our contribution is three-folded: first, the results from the current study would contribute to the general field of research related to the unethical behavior in organizational environments; additionally, the instrument that we propose, i.e., an adapted version of Bandura et al.'s (1996) moral disengagement scale, may be used in future research that focuses on unethical organizational behavior, regardless of the work domain. Second, we address some of the contrasting results related to the relationship between age and moral disengagement behavior, aiming to expand this area's results. Furthermore, our paper's practical contribution lies within the importance of predicting immoral organizational behavior for the work climate and company management. Moreover, given the specific area that we focused on (i.e., IT organizations) and the implications of the related potential unethical conduct (i.e., dark web or deep web), it is all the more important to identify personal characteristics that may predict organizational moral disengagement.

## Method

### Participants and Procedure

One hundred and fourteen individuals voluntarily participated in the present research. Their age varied from 21 to 54 (*M* = 28.51, *SD* = 4.56), most of them being males (62.1%), with a Bachelor's degree (49%). Their work experience varied from 2 years (13.1%) to 34 years (.08%). They were all employees of private IT international companies, with Romanian offices in the country's capital. All participants were active IT workers at the time of the study, with work experience at their current workplace, varying from less than one year (.08%) to 8 years (1.6%).

Participants were selected using the LinkedIn platform. They all received an individual message with the invitation to participate in the current online study anonymously. They were informed that participation was voluntary and anonymous and may withdraw at any time from the study. Confidentiality was guaranteed, and the completion time was estimated to be around 15 minutes. The form comprised five sections: the first section contained the informed consent, and the last one asking for demographic data (i.e., age, gender, education, IT work experience, experience at the current workplace). The other sections consisted of the Machiavellianism and psychopathy measurements and the organizational moral disengagement scale. The study was presented as part of a more extensive study on the Romanian organizational IT environment.

### Measures

We used the two subscales from The Short Dark Triad (SD3, Jones & Paulhus, 2014) to measure Machiavellianism and psychopathy. The Machiavellianism scale contained nine items (e.g., "Whatever it takes, you must get the important people on your side"; "There are things you should hide from other people to preserve your reputation"), participants answering on a 5-point Likert scale, from 1 (strongly disagree) to 5 (strongly agree). Similarly, the psychopathy scale contained nine items, such as "Payback needs to be quick and nasty" or "People who mess with me always regret it," on the same 5-point scale.

We used an adapted version (to the organizational environment) of Bandura et al.'s (1996) moral disengagement scale. Describing the same eight mechanisms proposed by the authors, we developed a similar instrument, containing 32 items (as in Bandura et al.'s original version of the scale), four for each moral disengagement dimension (*moral justification, advantageous comparison, euphemistic labeling, advantageous comparison, displacement of responsibility, diffusion of responsibility, disregard or distortion of consequences, dehumanization, and attribution of blame)*. Participants answered on a 5-point Likert scale, from 1 (strongly disagree) to 5 (strongly agree), a lower score suggesting a lower organizational moral disengagement level. Example items include: *It is all right to fight to protect your co-workers* (moral justification); *Slapping and shoving a colleague at work is just a way of joking* (euphemistic labeling); *Stealing some money from work is not too serious compared to those who steal a lot of money* (advantageous comparison); *Employees cannot be blamed for using bad words when all their co-workers do it* (displacement of responsibility); *If a group of co-workers decides together to do something harmful, it is unfair to blame any employee in the group for it* (diffusion of responsibility); *Teasing a co-worker does not really hurt them* (distorting consequences); *If employees fight and misbehave at work it is their leader's fault* (attribution of blame); *Some employees have to be treated roughly because they lack feelings that can be hurt* (dehumanization). Cronbach's alpha indicated a satisfying value of .865 for the scale.

We followed the back-forward translation method for all scales and pretested them in a sample of 27 individuals aged 20 to 42 (*M* = 27.92, *SD* = 5.71), 70.4% females. No issues were found within the items of the scales in the translation procedure, and all instruments provided satisfying internal consistency values (Cronbach's alphas > .70). All instruments were self-report questionnaires.

## Results

We used the 20.0 version of the SPSS program to analyze our data. We first conducted a series of preliminary analysis before computing a multiple linear hierarchical regression. First, we tested for multi-collinearities, results indicating that the variance inflation factor (VIF) values were all within the acceptable limits. Next, we analyzed the residual and scatter plots (Pallant, 2002) and verified the homoscedasticity condition. We then explored the associations between the variables we included in our prediction model (see Table 1). Results suggested a significant association with gender (*r* = -.253, *p* = .007) and age (*r* = -.185, *p* = .049), as well as with Machiavellianism (*r* = .551, *p* < .001) and psychopathy (*r* = .363, *p* < .001). In other words, younger males, with higher scores on both the Machiavellianism and psychopathy measures, scored higher on the organizational moral disengagement scale (see Table 1).

**Table 1:** Means, Standard Deviation, and Pearson Correlation Matrix for the Main Variables (N = 114)

Variable	*M*	*SD*	1	2	3	4	5	6	7	8
1. Gender			1							
2. Age	28.42	4.58	.082	1						
3. Education	4.16	0.65	.087	.148	1					
4. IT experience	6.56	4.60	.053	.915**	.104	1				
5. current workplace	2.15	1.58	.112	.099	.137	.086	1			
6. Moral Disengagement	55.31	11.62	-.253**	-.185*	-.032	-.169	.109	1		
7. Machiavellianism	25.37	6.13	-.181	-.013	-.098	.029	.006	.551**	1	
8. Psychopathy	20.38	4.89	-.199*	-.031	-.118	-.017	-.057	.363**	.462**	1

*p < .05. **p < .001.

We then performed a four-step linear hierarchical regression, with gender and age (step 1), total work experience and current work experience (step 2), Machiavellianism (step 3), and psychopathy (step 4) as predictors for organizational moral disengagement. Regression statistics are presented in Table 2.

**Table 2:** Summary of Hierarchical Regression Analysis for Variables Predicting Organizational Moral Disengagement (N = 114)

	Model 1			Model 2			Model 3			Model 4		
Variables	*B*	*SE B*	β	*B*	*SE(B)*	β	*B*	*SE B*	β	*B*	*SE B*	β
Gender	-6.06	2.29	-.241*	-6.45	2.30	-.257*	-4.27	1.95	-.170*	-0.391	1.96	-.156*
Age	-0.412	0.232	-.162	-0.375	0.573	-.147	-0.074	0.483	-.029	-0.091	0.481	-.036
IT work experience				-0.081	0.567	-.032	-0.392	0.478	-.155	-0.372	0.477	-.147
Current workplace				1.13	0.672	.155	1.01	0.564	.138	1.058	0.563	.144
Machiavellianism							1.00	0.148	.527**	0.907	0.164	.475**
Psychopathy										0.280	0.206	.117
*R^2^*	.092			.115			.383			.394		
*F* for change in *R^2^*	5.55*			1.44			46.42**			1.85		

*p < .05. **p < .001.

The hierarchical multiple regression revealed that at stage one, age and gender significantly contributed to the regression model, *F* (2, 112) = 5.55, *p* = .005) and accounted for 7.5% of the variation in organizational moral disengagement. Introducing the total work experience and current work experience explained an additional 1.8% of the dependent variable variation, but this change in R^2^ was not significant, *F* (2, 108) = 1.44, *p* = .241. Adding Machiavellianism to the regression model explained an additional 27.1% of the variation in organizational moral disengagement, and this change in *R*^2^ was significant: *F* (1, 107) = 46.42, *p* < .001. The psychopathy measure added at stage 4 explained an additional 0.5% of the organizational moral disengagement variation, but this change in R^2^ was not significant, *F* (1, 106) = 1.85, *p* = .177. When all six independent variables were included in stage four of the regression model, the most significant predictor of organizational moral disengagement was Machiavellianism (β = .475), followed by gender (β = -.156). Together, the six independent variables accounted for 35.9% of the variance in organizational moral disengagement.

For a deeper understanding of the present results, we explored the associations between all eight dimensions of moral disengagement, Machiavellianism, and psychopathy. Machiavellianism significantly correlated with all eight dimensions, and the most powerful correlation found was with the moral justification dimension (*r* = .496, *p* < .001). Similarly, psychopathy also correlated with most moral disengagement dimensions, except diffusion of responsibility (*r* = .159, *p* = .093) and dehumanization. The most powerful association with psychopathy, among the eight moral disengagement mechanisms, was found to be with euphemistic language (*r* = .319, *p* = .001) (see Table 3).

**Table 3:** Correlation Matrix Between the Main Variables and MD Mechanisms

Variable	1	2	3	4	5	6	7	8	9	10
1. Machiavellianism	1									
2. Psychopathy	.462**	1								
3. Moral justification	.496**	.287**	1							
4. Euphemistic language	.329**	.319**	.382**	1						
5. Advantageous comparison	.288**	.184	.196*	.426**	1					
6. Displacement of responsibility	.398**	.278**	.361**	.448**	.375**	1				
7. Diffusion of responsibility	.265**	.159	.286**	.241**	.343**	.355**	1			
8. Distorting consequences	.400**	.208*	.291**	.510**	.399**	.504**	.257**	1		
9. Attribution of blame	.340**	.311**	.228*	.118	.256**	.467**	.341**	.306**	1	
10. Dehumanization	.406**	.181	.342**	.452**	.453**	.473**	.288**	.430**	.350**	1

*p < .005. **p < .001.

## Discussion

In a cross-sectional study among IT workers aged 21 to 54, a hierarchical regression analysis suggested that the most important predictor of organizational moral disengagement was Machiavellianism, followed by gender. We assumed that all the other considered predictors in our final prediction model would be found significant (i.e., age, total work experience, current workplace experience, psychopathy), but no other significant predictors were found.

A significant, negative association emerged within the correlational analysis between organizational moral disengagement and age, confirming previous findings that suggest that the older we grow, the lower the moral disengagement (Paciello, Fida, Tramontano, Lupinetti, & Caprara, 2008). However, this result has been contradicted by several other studies (e.g., Roozen, De Pelsmacker, & Bostyn, 2001), and further studies are needed to clarify this specific association. More importantly, though significantly associated with organizational moral disengagement, age was not a significant predictor in our model. We also found that males are more likely to morally disengage within the organizational environment compared to females, confirming previous research related to gender differences in moral disengagement (Chonko & Hunt, 1985; Clemente, Espinosa, & Padilla, 2019; Fritzsche, 1988; Samnani et al., 2014; Wang et al., 2016).

Machiavellianism was the most powerful predictor in our final model, confirming the strongest correlation we previously found with organizational moral disengagement. Our results confirm the significant link between unethical behavior and the associated behaviors expressed by individuals high in Machiavellianism, such as spreading rumors about their co-workers, hiding important work information, or denigrating other co-workers (Czibor, 2012; Greenberg & Baron, 2003; Levashina & Campion, 2006; Roulin & Bourdage, 2017).

Unsurprisingly, we found that Machiavellianism and psychopathy were significantly associated with all moral disengagement mechanisms (with one exception, namely psychopathy and diffusion of responsibility). The most powerful association we found between the eight mechanisms and the two dimensions of the dark triad were between Machiavellianism and moral justification and between psychopathy and euphemistic language. In the process of moral justification, people can act upon presumed moral imperatives that are used to justify immoral acts. According to Bandura (1999), through the "moral justification of violent means, people see themselves as fighting ruthless oppressors, protecting their cherished values, preserving world peace, saving humanity from subjugation, or honoring their country's commitments" (p. 195). Within the organizational environment, using moral justification may be expressed by individuals who justify their immoral behavior by considering their unethical conduct as a way of "saving," for example, other co-workers from managers' exploitation. One may use other reasons, such as the good of the company, or the good of team-workers to wrap unethical deeds morally. Our results suggest that high levels of Machiavellianism, as well as psychopathy, are significantly associated with such cognitive mechanisms, which is not surprising, given previous data that describes such individuals as manipulative, cynical, secretive, opportunistic, suspicious (Christie & Geis, 1970; Ináncsi, Láng, & Bereczkei, 2015; 2016; Jaffé, Greifeneder, & Reinhard, 2019), and lacking empathy (Ali, Amorim, and Chamorro-Premuzic, 2009).

Euphemistic language is generally used to make harmful, unethical behavior look less negative and lower one's responsibility for an immoral act. According to Bandura (1999), "by camouflaging pernicious activities in innocent or sanitizing parlance, the activities lose much of their repugnancy" (p. 195). For example, civilians killed during bomb-attacks are presented as "collateral damage," pornography is portrayed as a form of cheap sex, and fired employees are "let go." Other frequently used forms of sanitized language within the organizational environment are words and expressions such as "economically disadvantaged" instead of poor, "compensation plan" instead of "pay," "to downsize," instead of "to fire." The significant association with both psychopathy and Machiavellianism is unsurprising, given their general characteristics.

In line with previous findings (e.g., Roozen, De Pelsmacker, & Bostyn, 2001), we assumed that participants with limited work experience would score lower on the organizational moral disengagement scale. Our results did not confirm any associations between the general work experience nor one's experience at the current workplace. Instead, results confirmed other personal factors (i.e., gender, age, Machiavellianism, and psychopathy) as significantly correlated with organizational moral disengagement and its mechanisms. Therefore, it seems that the organizational environment itself and the organizational culture seem to matter less when it comes to moral disengagement within one's workplace. In other words, personal characteristics, both demographic and those related to personality, were much stronger predictors and associated factors compared to the correlates of the organizational environment. This result is all the more important as it suggests that those responsible for monitoring and preventing unethical behaviors in the workplace should consider the individual himself, formulate strategies taking into account his or her individual characteristics, and concentrate less on organizational policies.

Though we used a novel approach to measure organizational moral disengagement, namely a personalized scale, adapted from Bandura et al. 's (1996) well-known scale, our instrument did not specifically measure the IT environment. Therefore, our results are not highly generalizable for several reasons: on the one hand, due to the small number of participants, and on the other hand, because the scale used has not yet been validated nor specifically adapted to the IT industry. Additionally, causality, common method bias, and the sample's diverse nature may also be considered important limitations for the current research. Therefore, future studies might want to benefit from using experimental and longitudinal approaches to address such issues and limit social desirability or consistency motif (Podsakoff, MacKenzie, & Podsakoff, 2012). However, despite these limitations, we consider the present study results as an important starting point for future studies that will try to define and establish the most significant predictors of moral disengagement in the organizational environment.

There are several theoretical and practical implications for the present research. First, our results add to the data related to the link between Machiavellianism, psychopathy, and moral disengagement at the organizational level. Second, our results confirm a series of studies related to the associations between age and moral disengagement, which, in turn, contradict other results in the area. On the other hand, the associations between the dimensions of organizational moral disengagement and psychopathy, respectively, Machiavellianism, can be beneficial for organizations fighting unethical behaviors in the workplace. At a more practical level, our result highlighted the importance of organizational moral education programs, such as collective and group ethically focused trainings. More specifically, our findings suggest that human resources managers and organizational leaders should consider employees' personal traits when shaping effective strategies for monitoring and preventing unethical behaviors in the workplace.

## References

[r1] Ali, F., Amorim, I. S., & Chamorro-Premuzic, T. (2009). Empathy deficits and trait emotional intelligence in psychopathy and Machiavellianism. Personality and Individual Differences, 47(7), 758–762. 10.1016/j.paid.2009.06.016

[r2] Austin, E. F. (2007). Emotional intelligence, Machiavellianism and emotional manipulation: Does EI have a dark side? Personality and Individual Differences, 43(1), 179–189. 10.1016/j.paid.2006.11.019

[r3] Bandura, A. (1971). *Social learning theory.* Prentice-Hall.

[r4] Bandura, A. (1999). Moral disengagement in the perpetration of inhumanities. Personality and Social Psychology Review*,* 3(3), 193–209. 15661671 10.1207/s15327957pspr0303_3

[r5] Bandura, A. (2002). Selective moral disengagement in the exercise of moral agency. Journal of Moral Education, 31(2), 101–119. 10.1080/0305724022014322

[r6] Bandura, A., Barbaranelli, C., Caprara, G., & Pastorelli, C. (1996). Mechanisms of moral disengagement in the exercise of moral agency. Journal of Personality and Social Psychology, 71(2), 364–374. 10.1037/0022-3514.71.2.364

[r7] Barbaranelli C., & Perna A. (2004). Meccanismi di disimpegno morale nell'applicazione delle normative sulla sicurezza: Contributo empirico [Mechanisms of moral disengagement and safety in the workplace: An empirical contribution]. Risorsa Uomo: Rivista di Psicologia del Lavoro e dell'Organizzazione*,* 10(4), 393–415.

[r8] Barsky, A. (2011). Investigating the effects of moral disengagement and participation on unethical work behavior. Journal of Business Ethics, 104(1), 59–75. 10.1007/s10551-011-0889-7

[r10] Bjärehed, M. T. (2020). Mechanisms of moral disengagement and their associations with indirect bullying, direct bullying, and pro- aggressive bystander behavior. The Journal of Early Adolescence, 40(1), 28–55. 10.1177/0272431618824745

[r11] Boddy, C. (2005). The implications of corporate psychopaths for business and society: An initial examination and a call to arms. Australasian Journal of Business and Behavioral Sciences, 1(2), 30–40.

[r12] Brewer, G., & Abell, L. (2017). Machiavellianism, relationship satisfaction, and romantic relationship quality. Europe’s Journal of Psychology, 13(3), 491–502. 10.5964/ejop.v13i3.1217 28904597 PMC5590532

[r13] Chonko, L. B., & Hunt, S. D. (1985). Ethics and marketing management: An empirical examination. Journal of Business Research, 13(4), 339–359. 10.1016/0148-2963(85)90006-2

[r14] Christie, R., & Geis, F. L. (1970). Studies in Machiavellianism. Academic Press.

[r15] Clemente, M., Espinosa, P., & Padilla, D. (2019). Moral disengagement and willingness to behave unethically against ex-partner in a child custody dispute. PLoS One, 14(3), e0213662. 10.1371/journal.pone.0213662 30865710 PMC6415824

[r16] Cohen, A. (2016). Are they among us? A conceptual framework of the relationship between the dark triad personality and counterproductive work behaviors (CWBs). Human Resource Management Review, 26(1), 69–85. 10.1016/j.hrmr.2015.07.003

[r17] Czibor, A. B. (2012). Machiavellian people’s success results from monitoring their partners. Personality and Individual Differences, 53(3), 202–206. 10.1016/j.paid.2012.03.005

[r18] D’Souza, M. F. (2015). The dark side of power: The dark triad in opportunistic decision making. Advances in Scientific and Applied Accounting, 8(2), 135–156. 10.14392/asaa.2015080201

[r19] Egan, V., Hughes, N., & Palmer, E. J. (2015). Moral disengagement, the dark triad, and unethical consumer attitudes. Personality and Individual Differences, 76, 123–128. 10.1016/j.paid.2014.11.054

[r20] Ernst & Young. (2017). Human instinct, machine logic: Which do you trust most in the fight against fraud and corruption? https://assets.ey.com/content/dam/ey-sites/ey-com/en_gl/topics/digital/ey-emeia-fraud-survey-2017.pdf

[r21] Eurostat. (2017). ICT specialists. More than 8 million ICT specialists employed in the EU in 2016. A largely male and highly educated workforce. https://ec.europa.eu/eurostat/documents/2995521/8115840/9-18072017-AP-EN.pdf/b775e424-a14c-4037-9b33-5cc97164bc11

[r22] Fida, R., Paciello, M., Tramontano, C., Fontaine, R. G., Barbaranelli, C., & Farnese, M. L. (2015). An integrative approach to understanding counterproductive work behavior: The roles of stressors, negative emotions, and moral disengagement. Journal of Business Ethics, 130(1), 131–144. 10.1007/s10551-014-2209-5

[r23] Fritzsche, D. J. (1988). An examination of marketing ethics: Role of the decision maker, consequences of the decision, management position and sex of respondent. Journal of Macromarketing, 8(2), 29–39. 10.1177/027614678800800205

[r24] Glenn, A. L., & Sellbom, M. (2015). Theoretical and empirical concerns regarding the dark triad as a construct. Journal of Personality Disorders, 29(3), 360–377. 10.1521/pedi_2014_28_162 25248015

[r25] Greenberg, J., & Baron, R. A. (2003). *Behaviour in organization. Understanding and managing the human side of work*. Prentice Hall.

[r26] Greenberg, J., & Baron, R. A. (2013). *Behavior in organizations: Understanding and managing the human side of work* (4th ed.). Allyn & Bacon.

[r27] Hogan, J., Hogan, R., & Kaiser, R. B. (2011). Management derailment. In S. Zedeck (Ed.), *APA handbook of industrial and organizational psychology* (Vol. 3, pp. 555–576). APA Books.

[r28] Huang, G.-H., Wellman, N., Ashford, S. J., Lee, C., & Wang, L. (2017). Deviance and exit: The organizational costs of job insecurity and moral disengagement. The Journal of Applied Psychology, 102(1), 26–42. 10.1037/apl0000158 27618406

[r29] Ináncsi, T., Láng, A., & Bereczkei, T. (2015). Machiavellianism and adult attachment in general interpersonal relationships and close relationships. Europe’s Journal of Psychology, 11(1), 139–154. 10.5964/ejop.v11i1.801 27247647 PMC4873099

[r30] Ináncsi, T., Láng, A., & Bereczkei, T. (2016). A darker shade of love: Machiavellianism and positive assortative mating based on romantic ideals. Europe’s Journal of Psychology, 12(1), 137–152. 10.5964/ejop.v12i1.1007 27247697 PMC4873071

[r31] Jaffé, M. E., Greifeneder, R., & Reinhard, M. A. (2019). Manipulating the odds: The effects of Machiavellianism and construal level on cheating behavior. PLoS One, 14(11), e0224526. 10.1371/journal.pone.0224526 31725739 PMC6855464

[r32] Jonason, P. K., Li, N. P., & Czarna, A. Z. (2013). Quick and dirty: Some psychosocial costs associated with the dark triad in three countries. Evolutionary Psychology, 11(1), 172–185. 10.1177/14747049130110011623531804

[r33] Jonason, P. K., Webster, G. D., Schmitt, D. P., Li, N. P., & Crysel, L. (2012). The antihero in popular culture: Life history theory and the dark triad personality traits. Review of General Psychology, 16(2), 192–199. 10.1037/a0027914

[r34] Jones, D. F. (2013). The core of darkness: Uncovering the heart of the dark triad. European Journal of Personality, 27(6), 521–531. 10.1002/per.1893

[r35] Jones, D. N., & Paulhus, D. L. (2014). Introducing the short dark triad (SD3): A brief measure of dark personality traits. Assessment, 21(1), 28–41. 10.1177/1073191113514105 24322012

[r36] Kaufman, S. B., Yaden, D. B., Hyde, E., & Tsukayama, E. (2019). The light vs. dark triad of personality: Contrasting two very different profiles of human nature. Frontiers in Psychology, 10 Article e467. 10.3389/fpsyg.2019.00467 30914993 PMC6423069

[r37] LeBreton, J. M., Binning, J. F., & Adorno, A. J. (2006). Subclinical psychopaths. In D. L. Segal & J. C. Thomas (Eds.), *Comprehensive handbook of personality and psychopathology* (Vol. 1, pp. 388–411). Wiley.

[r38] Levashina, J., & Campion, M. A. (2006). A model of faking likelihood in the employment interview. International Journal of Selection and Assessment, 14(4), 299–316. 10.1111/j.1468-2389.2006.00353.x

[r39] Lyons, M. (2019). *The dark triad of personality. Narcissism, Machiavellianism, and psychopathy in everyday life*. Elsevier

[r40] Lyons, M., Houghton, E., Brewer, G., & O’Brien, F. (2022). The dark triad and sexual assertiveness predict sexual coercion differently in men and women. Journal of Interpersonal Violence, 37(7–8), NP4889–NP4904. 10.1177/0886260520922346 32438885 PMC8980444

[r41] Martin, S. R., Kish-Gephart, J. J., & Detert, J. R. (2014). Blind forces: Ethical infrastructures and moral disengagement in organizations. Organizational Psychology Review, 4(4), 295–325. 10.1177/2041386613518576

[r42] Mathieu, C., Neumann, C., Babiak, P., & Hare, R. D. (2015). Corporate psychopathy and the full-range leadership model. Assessment, 22(3), 267–278. 10.1177/1073191114545490 25092715

[r43] Mealey, L. (1995). The sociobiology of sociopathy: An integrated evolutionary model. Behavioral and Brain Sciences, 18(3), 523–541. 10.1017/S0140525X00039595

[r44] Moore, C. (2008). Moral disengagement in processes of organizational corruption. Journal of Business Ethics, 80(1), 129–139. 10.1007/s10551-007-9447-8

[r45] Moore, C. D. (2012). Why employees do bad things: Moral disengagement and unethical organizational behavior. Personnel Psychology, 65(1), 1–48. 10.1111/j.1744-6570.2011.01237.x

[r46] Moraga, F. R., Nima, A. A., & Garcia, D. (2017). Sex and dark times’ strategy: The dark triad and time perspective. PsyCh Journal, 6(1), 98–99. 10.1002/pchj.153 28105766

[r47] Nübold, A., Bader, J., Bozin, N., Depala, R., Eidast, H., Johannessen, E. A., & Prinz, G. (2017). Developing a taxonomy of dark triad triggers at work—A grounded theory study protocol. Frontiers in Psychology, 8 Article e293. 10.3389/fpsyg.2017.00293 28326048 PMC5339219

[r48] O’Boyle, E. H., Jr., Forsyth, D. R., Banks, G. C., & McDaniel, M. A. (2012). A meta-analysis of the dark triad and work behavior: A social exchange perspective. The Journal of Applied Psychology, 97(3), 557–579. 10.1037/a0025679 22023075

[r49] Paciello, M., Fida, R., Tramontano, C., Lupinetti, C., & Caprara, G. (2008). Stability and change of moral disengagement and its impact on aggression and violence in late adolescence. Child Development, 79(5), 1288–1309. 10.1111/j.1467-8624.2008.01189.x 18826526

[r50] Pallant, J. (2002). *SPSS survival manual. A step by step guide to data analysis using IBM SPSS*. Routledge. 10.4324/9781003117452 10.4324/9781003117452

[r51] Palmer, J. K., Komarraju, M., Carter, M. Z., & Karau, S. J. (2017). Angel on one shoulder: Can perceived organizational support moderate the relationship between the dark triad traits and counterproductive work behavior? Personality and Individual Differences, 110, 31–37. 10.1016/j.paid.2017.01.018

[r52] Paulhus, D. L., & Williams, K. M. (2002). The dark triad of personality: Narcissism, Machiavellianism, and psychopathy. Journal of Research in Personality, 36(6), 556–563. 10.1016/S0092-6566(02)00505-6

[r53] Pavlov, I. (1926). *Conditioned reflexes.* Dover Publications

[r54] Pilch, I., & Turska, E. (2015). Relationships between Machiavellianism, organizational culture, and workplace bullying: Emotional abuse from the target’s and the perpetrator’s perspective. Journal of Business Ethics, 128(1), 83–93. 10.1007/s10551-014-2081-3

[r55] Podsakoff, P. M., MacKenzie, S. B., & Podsakoff, N. P. (2012). Sources of method bias in social science research and recommendations on how to control it. Annual Review of Psychology, 63, 539–569. 10.1146/annurev-psych-120710-100452 21838546

[r56] Prusik, M., & Szulawski, M. (2019). The relationship between the dark triad personality traits, motivation at work, and burnout among HR recruitment workers. Frontiers in Psychology, 10 Article e1290. 10.3389/fpsyg.2019.01290 31231283 PMC6566539

[r57] Qin, X., Shepherd, D. A., Lin, D., Xie, S., Liang, X., & Lin, S. (2020). The dark side of entrepreneurs’ creativity: Investigating how and when entrepreneurs’ creativity increases the favorability of potential opportunities that harm nature. Entrepreneurship Theory and Practice, 4, e104225872091558. Advance online publication. 10.1177/1042258720915582

[r58] Roeser, K., McGregor, V. E., Stegmaier, S., Mathew, J., Kübler, A., & Meule, A. (2016). The dark triad of personality and unethical behavior at different times of day. Personality and Individual Differences, 88, 73–77. 10.1016/j.paid.2015.09.002

[r59] Roozen, I., De Pelsmacker, P., & Bostyn, F. (2001). The ethical dimensions of decision processes of employees. Journal of Business Ethics, 33(2), 87–99. 10.1023/A:101753622235511837286

[r60] Roulin, N., & Bourdage, J. S. (2017). Once an impression manager, always an impression manager? Antecedents of honest and deceptive impression management use and variability across multiple job interviews. Frontiers in Psychology, 8 Article e29. 10.3389/fpsyg.2017.00029 28174546 PMC5258756

[r61] Sabouri, S., Gerber, M., Sadeghi Bahmani, D., Lemola, S., Clough, P. J., Kalak, N., Shamsi, M., Holsboer-Trachsler, E., & Brand, S. (2016). Examining dark triad traits in relation to mental toughness and physical activity in young adults. Neuropsychiatric Disease and Treatment, 12, 229–235. 10.2147/NDT.S97267 26869790 PMC4737324

[r62] Samnani, A., Salamon, S. D., & Singh, P. (2014). Negative affect and counterproductive workplace behavior: The moderating role of moral disengagement and gender. Journal of Business Ethics, 119(2), 235–244. 10.1007/s10551-013-1635-0

[r63] Schyns, B. (2015). Dark personality in the workplace: Introduction to the special issue. Applied Psychology, 64(1), 1–14. 10.1111/apps.1204125820206

[r64] Sijtsema, J. J., Garofalo, C., Jansen, K., & Klimstra, T. A. (2019). Disengaging from evil: Longitudinal associations between the dark triad, moral disengagement, and antisocial behavior in adolescence. Journal of Abnormal Child Psychology, 47(8), 1351–1365. 10.1007/s10802-019-00519-4 30737660 PMC6617551

[r65] Skinner, B. F. (1966). *The behavior of organisms: An experimental analysis*. Appleton-Century.

[r66] Spurk, D., Keller, A. C., & Hirschi, A. (2016). Do bad guys get ahead or fall behind? Relationships of the dark triad of personality with objective and subjective career success. Social Psychological & Personality Science, 7(2), 113–121. 10.1177/1948550615609735

[r67] Szabó, E., & Bereczkei, T. (2017). Different paths to different strategies? Unique associations among facets of the dark triad, empathy, and trait emotional intelligence. Advances in Cognitive Psychology, 13(4), 306–313. 10.5709/acp-0230-7 29362646 PMC5771371

[r68] ten Brinke, L., Kish, A., & Keltner, D. (2018). Hedge fund managers with psychopathic tendencies make for worse investors. Personality and Social Psychology Bulletin, 44(2), 214–223. 10.1177/0146167217733080 29047319

[r69] Thorndike, E. (1898). Animal intelligence: An experimental study of the associative processes in animals. The Psychological Review: Monograph Supplements, 2(4), i–109. 10.1037/10780-000

[r70] Tolman, E. C. (1932). *Purposive behavior in animals and men.* Century/Random House.

[r72] Wang, C., Ryoo, J. H., Swearer, S. M., Turner, R., & Goldberg, T. S. (2017). Longitudinal relationships between bullying and moral disengagement among adolescents. Journal of Youth and Adolescence, 46(6), 1304–1317. 10.1007/s10964-016-0577-0 27704302

[r71] Wang, X., Lei, L., Liu, D., & Hu, H. (2016). Moderating effects of moral reasoning and gender on the relation between moral disengagement and cyberbullying in adolescents. Personality and Individual Differences, 98, 244–249. 10.1016/j.paid.2016.04.056

[r73] Wissing, B. G., & Reinhard, M.-A. (2019). The dark triad and deception perceptions. Frontiers in Psychology, 10 Article e1811. 10.3389/fpsyg.2019.01811 31456714 PMC6700213

[r74] Wu, W., Wang, H., Zheng, C., & Wu, Y. J. (2019). Effect of narcissism, psychopathy, and Machiavellianism on entrepreneurial intention—The mediating of entrepreneurial self-efficacy. Frontiers in Psychology, 10 Article e360. 10.3389/fpsyg.2019.00360 30846958 PMC6393355

